# Systematic evaluation and optimization of protein extraction parameters in diagnostic FFPE specimens

**DOI:** 10.1186/s12014-022-09346-0

**Published:** 2022-05-02

**Authors:** Franz F. Dressler, Jana Schoenfeld, Olga Revyakina, Daniel Vogele, Selina Kiefer, Jutta Kirfel, Timo Gemoll, Sven Perner

**Affiliations:** 1 Institute of Pathology, University Medical Center Schleswig-Holstein, Luebeck Site, Ratzeburger Allee 160, 23562 Luebeck, Germany; 2 Center for Biological Systems Analysis, Core Facility Proteomics, University of Freiburg, Habsburgerstraße 49, 79104 Freiburg, Germany; 3 Department of Pathology, Medical Center - University of Freiburg, Faculty of Medicine, University of Freiburg, Freiburg, Germany; 4 Section for Translational Surgical Oncology and Biobanking, Department of Surgery, University Medical Center Schleswig-Holstein, Campus Luebeck, Ratzeburger Allee 160, 23562 Luebeck, Germany; 5 Institute of Pathology, Research Center Borstel, Leibniz Lung Center, Parkallee 1-40, 23845 Borstel, Germany; 6 Institute of Pathology, Charité - Universitätsmedizin Berlin, corporate member of Freie Universität Berlin, Humboldt-Universität zu Berlin, and Berlin Institute of Health, Berlin, Germany

**Keywords:** Protein extraction, Formalin-fixed paraffin-embedded tissue, Clinical proteomics, Antigen retrieval, Crosslinking reversal, Protein analysis, Biomarker

## Abstract

**Objectives:**

Formalin-fixed paraffin-embedded (FFPE) tissue is the standard material for diagnostic pathology but poses relevant hurdles to accurate protein extraction due to cross-linking and chemical alterations. While numerous extraction protocols and chemicals have been described, systematic comparative analyses are limited. Various parameters were thus investigated in their qualitative and quantitative effects on protein extraction (PE) efficacy. Special emphasis was put on preservation of membrane proteins (MP) as key subgroup of functionally relevant proteins.

**Methods:**

Using the example of urothelial carcinoma, FFPE tissue sections were subjected to various deparaffinization, protein extraction and antigen retrieval protocols and buffers as well as different extraction techniques. Performance was measured by protein concentration and western blot analysis of cellular compartment markers as well as liquid chromatography-coupled mass spectrometry (LC–MS).

**Results:**

Commercially available extraction buffers showed reduced extraction of MPs and came at considerably increased costs. On-slide extraction did not improve PE whereas several other preanalytical steps could be simplified. Systematic variation of temperature and exposure duration demonstrated a quantitatively relevant corridor of optimal antigen retrieval.

**Conclusions:**

Preanalytical protein extraction can be optimized at various levels to improve unbiased protein extraction and to reduce time and costs.

**Supplementary Information:**

The online version contains supplementary material available at 10.1186/s12014-022-09346-0.

## Introduction

In spite of recent technical advances in bottom-up proteomics, data quality and translational usability still hinge on accurate preanalytical sample preparation. Other omics levels such as genome and transcriptome intrinsically allow amplification of minute amounts of starting material, which is not possible for proteins. Optimal quantitative retrieval of proteins from any source material is thus vital but hampered by the molecular heterogeneity of proteins. With the chemical and physical behavior already hard to predict on single-molecule level, mixtures of thousands of proteins (as typically found in tissue and cell extracts) prove to be of even higher complexity.

It is against this complicated background that formalin treatment of medical specimens adds further modifications and crosslinks. Formalin is necessary to increase tissue rigidity sufficiently for diagnostic sections and to conserve both macro- and microstructure of the organic material. It leads to diverse crosslinks between proteins and other macromolecules [1–3], mostly involving amino and thiol groups of lysine and cysteine but many other functional groups as well [4]. Formalin-fixed, dehydrated and paraffin-embedded tissue (FFPE) offers optimal conditions for histomorphological diagnostics, which is also relevant for translational biomedical research to allow for accurate identification of target (e.g. tumor) cells by microdissection. For these reasons, well-characterized tissue archives with clinical follow-up data for diagnostic and research purposes comprise almost exclusively FFPE tissue. These archives in turn form the mainstay of biological information available to decipher and ultimately treat human diseases.

While RNA and DNA are comparatively straightforward to analyze—even from FFPE specimens—their alterations bear only indirect relation to protein changes [5]. The latter, however, carry the vast majority of cellular functions in both healthy and diseased states. They are the predominant target of drugs and therapeutics [6]. Routine diagnostic measurement of protein expression from FFPE material has so far only been established by immunohistochemical (IHC) methods but suffers from a lack of accurate quantitation [7]. As a potential alternative, mass spectrometry-based quantitation in turn depends on highly standardized and reproducible protein extraction.

Based on the importance of FFPE tissue, numerous studies have investigated the effects of optimized sample preparation on protein extraction (PE) efficacy. First, the paraffin wax has to be removed. Both organic solvents such as xylene and heptane as well as simple thermal melting have been described [8–10] but their effects on PE and especially preservation of membrane proteins (MP) were incompletely determined.

Second, formalin-induced crosslinks have to be reversed after rehydratation. The application of thermal energy has been recognized as a key element but several temperature regimes exist [11–14]. Also, the effects of scavenger molecules on formalin reversal have been investigated with a significant influence of scavenger concentration on PE efficiency [15]. These results were limited in terms of the investigated proteins and buffer combinations and were not corrected for ionic strength differences. Concerning the latter the general effects of cosmo- and chaotropic salts in complex protein extracts are far from trivial, in spite of century-long research [16]. Although previously investigated [17] little overlap of the chosen parameters exists with other publications [15], a general problem in the field of FFPE extraction optimization.

Last, the proteins have to be solubilized, for which detergents are a prime factor. In recent years particularly the removal of detergents incompatible with liquid-chromatography coupled mass spectrometry (LC–MS) has been improved [18–21]. However, the ideal choice of detergent for PE from FFPE remains a topic of ongoing investigation [13, 14, 18, 21–26]. Here too, parallel variation of extraction protocol, mode and buffer renders it difficult to separate optimization effects. Further evidence has been put forward to suggest that elevated pressure or direct on-slide extraction may be favorable for solubilization as well [24, 27–29].

In search for standardization commercially available kits are more and more used for archieval FFPE tissue [30–33], although considerable limitations concerning selective loss of extracted proteins have been reported [34]. The latter is particularly relevant for membrane proteins, which are difficult to extract and detect but are highly relevant in deciphering the pathogenesis of cancer and for the development of novel targeted therapies [35]. Against this background of broad yet patchy information the present study sets out for systematical variation and comparisons of key parameters.

## Materials and methods

### Input tissue

For all comparative analyses FFPE tissue from bladder cancer specimens was used as model solid tumor due to its interdigitating tumor growth between connective tissue bundles (Additional file 1: Fig. S1). The specimens were routine pathological samples, submitted to the Institute of Pathology, Luebeck, and cleared for research purposes after routine diagnostics by patient consent (ethics Committee University of Luebeck, vote 19-234). Fixation occurred according to diagnostic standardized operating procedures. FFPE material was stored up to 24 months until extraction. Standard serial sections of the same tissue block (10 µm thickness each) were alternatingly distributed onto the different experimental groups. Unless indicated otherwise, two sections were used in 106 µl extraction buffer.

### Buffers and chemicals

All buffers are listed in the Additional file 1: Table S2. Apart from the commercially available EXB buffer (buffer Com) as part of the qProteome FFPE kit (Qiagen 37623, Hilden, Germany), a buffer containing 0.1% (w/v) RapiGest (Waters 186001861, Eschborn, Germany) was prepared according to Foll et al. [36] (buffer RG) and modified by replacing HEPES with 200 mM Tris–HCl (buffer RG-T). Further extraction buffers were prepared with SDS and Zwittergent 3-16 (Santa Cruz BT 281194, Heidelberg, Germany): Buffer S containing SDS 8% (w/v), Tris-Base 200 mM; Buffer S-T containing SDS 8% (w/v), Tris-Base 10 mM; Buffer Z containing Zwittergent 2% (w/v), Tris-Base 200 mM; Buffer Z + S containing Zwittergent 2% (w/v) and SDS 8% (w/v). All custom buffers contained EDTA 1 mM and pH was titrated to 7.2 at room temperature with HCl. Before use, all buffers were supplemented with 5 µl beta-mercaptoethanol (Merck M6250, Darmstadt, Germany) and 1 µl proteinase and phosphatase inhibitor (ThermoFisher 78840, Dreieich, Germany) per 100 µl buffer. For pH variation a modified buffer S* was used containing SDS 2% (w/v) and Tris-Base 10 mM, adjusted to the respective pH with HCl (necessary amounts and thus ionic strength alterations were noted but varied only within 11.4 mM compared to an SDS concentration of 69 mM). For ionic strength variations the respective concentrations of NaCl were added.

Deparaffinization and rehydratation Initial thermal deparaffinization was performed according to Mansour et al. [9] with adaptation to 90 °C for three times of 2 min immersion in 15 ml tubes filled with prewarmed double-distilled water. For gentler deparaffinization (with theoretical preservation of biological membranes [37]) and parallel processing up to 10 slides were attached to the inner wall of a 2 L beaker filled with distilled water of 60 °C temperature, stirred at low speed for 30 min. Xylene deparaffinization was performed by incubation of whole slides in pure xylene for 15 min, repeated once. Rehydratation was performed by sequential immersion in ethanol for 10 min each with concentrations 100, 100 (repeat), 96 and 70% (v/v) respectively.

### Extraction modes

For temperature-varied extractions, a standard PCR thermocycler (Biometra T1, Gottingen, Germany) was used with 0.2 ml PCR tubes. On-slide extraction was performed using a commercially available PCR sealing system (Merck GBL611102). After deparaffinization, slides were cleaned with ethanol-wetted tissue wipers outside of the tissue area and the seal carefully placed onto the slide. Gentle pressure was applied, and the seal tightened by careful scratching with a blunt forceps handle. Buffer was easily infused to the reaction chamber by a standard pipet and capillary force. The openings were closed with the enclosed adhesive patches. After the heating cycles, the covers were incised with a scalpel, placed in 50 ml tubes and centrifuged at 1000*g* for 2 min to retrieve the buffer.

### SDS page and western blot analysis

Samples were prepared for SDS page in loading buffer (ThermoFisher NP0007) containing reducing agent (ThermoFisher NP0004) and run with MOPS buffer (ThermoFisher NP0050) in 15-well 10% pre-cast Bis–Tris gels (ThermoFisher NP0303BOX) for 35 min with current limits of 200 V and 250 mA. Samples were then blotted to nitrocellulose membranes with 0.45 µm pore size (ThermoFisher LC2001) in transfer buffer (ThermoFisher NP00061) at 30 V and 250 mA for 60 min. Membranes were cut and blocked in 20 ml PBS-T-M [PBS pH 7.4 (Merck P3813), Tween-20 0.5%, milk powder 5%] for 1 h. After washing two times with 20 ml PBS-T membranes were then incubated overnight at 4 °C on a rolling shaker in 6 ml PBS-T-M supplemented with the respective primary antibody. Membranes were then washed four times with 20 ml PBS-T for 3 min each and incubated at room temperature for 2 h in 20 ml PBS-T-M including the secondary antibody. Washing was repeated and membranes were visualized on a densitrometric imager (Amersham Imager 600, GE Healthcare 29083461, Freiburg, Germany) using ECL developing agents (GE Healthcare Life Sciences Europe RPN2106, Eindhoven, Netherlands). The respective primary antibodies and their dilutions were 1:1000 anti-Integrin beta1 (b-Integrin; monoclonal rabbit IgG; Cell Signaling 9699S, Leiden, Netherlands), 1:200 anti-ATP synthase F1 subunit beta (ATP5B; monoclonal mouse IgG; Santa-Cruz sc-74549); 1:100 anti-Epithelial cell adhesion molecule (EpCAM; monoclonal mouse IgG; Santa-Cruz sc-25308) and 1:100 anti-Hypoxanthine–guanine phosphoribosyltransferase (HPRT; monoclonal mouse IgG; Santa-Cruz sc-376938). Secondary antibodies were 1:2500 goat anti-rabbit IgG (ThermoFisher 31,460) and 1:2500 goat anti-mouse IgG (ThermoFisher 31,430). Prerequisites for relative protein quantitation were accurately followed as described by [38] and linear ranges determined beforehand.

### Protein concentration measurement

Concentrations were determined using the EZQ assay provided as a kit from ThermoFisher (R33200) according to the manufacturer’s instructions. Samples were measured directly and after fivefold dilution, each in duplicates. Membranes were visualized wet on a Gel Doc XR + imaging system (BioRad 1708195, Feldkirchen, Germany) using the build-in emission filter 1 and UV excitation (compatibility with the proprietary EZQ fluorophore was checked with the company). Spots on the resulting tiff image were quantified with ImageLab (version 6.1; BioRad 12012931) and converted into concentrations by standard linear regression in an Excel spreadsheet (version 16.39; Microsoft, Seattle, USA).

### Sample clean-up and tryptic digestion

For removal of detergents and digestion the qProteome protocol was followed including the methanol/chloroform precipitation according to the manufacturer’s instructions. For acetone precipitation the respective part of the protocol was substituted by the addition of four volumes of ice-cold acetone, freezing at −20 °C for 60 min and centrifugation at 10,000*g* and 4 °C for 10 min. Trypsin and DTT were purchased from Merck (T6567, P2325), iodoacetamide from BioRad [1, 632, 109]. For the deparaffinization and rehydratation experiments, digests were performed in-gel or in-solution using Stage tips (Additional file 1: Additional method S1). 10 fractions were analysed by each protocol.

### Mass spectrometric analysis

For liquid chromatography/tandem mass spectrometry (LC–MS) samples were lyophilized for 4 h and resuspended in Acteonitrile 2% (v/v) including formic acid 0.5% (v/v) to a final protein concentration of 1 µg/µl. Samples were then loaded onto a C18[2] column (15 cm, 3 µm Luna Phenomenex) in a Ultimate 3000 RSLCnano high-performance liquid chromatography system (HPLC; ThermoFisher) and injected on-line into a 5600 + TripleToF mass spectrometer (AB Sciex) in data-dependent mode with selection of 30 precursor ions. For the deparaffinization and rehydratation experiments samples were digested and fractionated [10 fractions] in-gel [39] or in-solution using Stage tips [40], separated using an Agilent 1200 series HPLC with NanoFlow pump and C18 column and analyzed on an LTQ-OrbiTrap XL mass spectrometer (ThermoFisher). Experiments were evaluated with MaxQuant 1.4.2.1 (1.0% false discovery rate (FDR); maximum two missed cleavages; minimum one unique peptide per identified protein; intensity above 0). For the final protocol comparison experiments were evaluated with ProteinPilot (version 5.0.2, AB Sciex, Darmstadt, Germany) at a local FDR of 5.0%. To evaluate the share of membrane proteins identified, subcellular localization information using the LOCATE database (version 6) [41] was appended to the dataset using a custom Python script. The mass spectrometry proteomics data have been deposited to the ProteomeXchange Consortium via the PRIDE [42] partner repository with the dataset identifier PXD029133.

### Statistical analyses and visualization

All statistical and descriptive analyses were performed in custom scripts in Python 2.7 (Enthought, Austin, USA, Canopy distribution 1.1.0.1371) including the scipy, numpy, matplotlib, seaborn and pandas packages.

## Results

### Evaluation setup

**Fig. 1:**
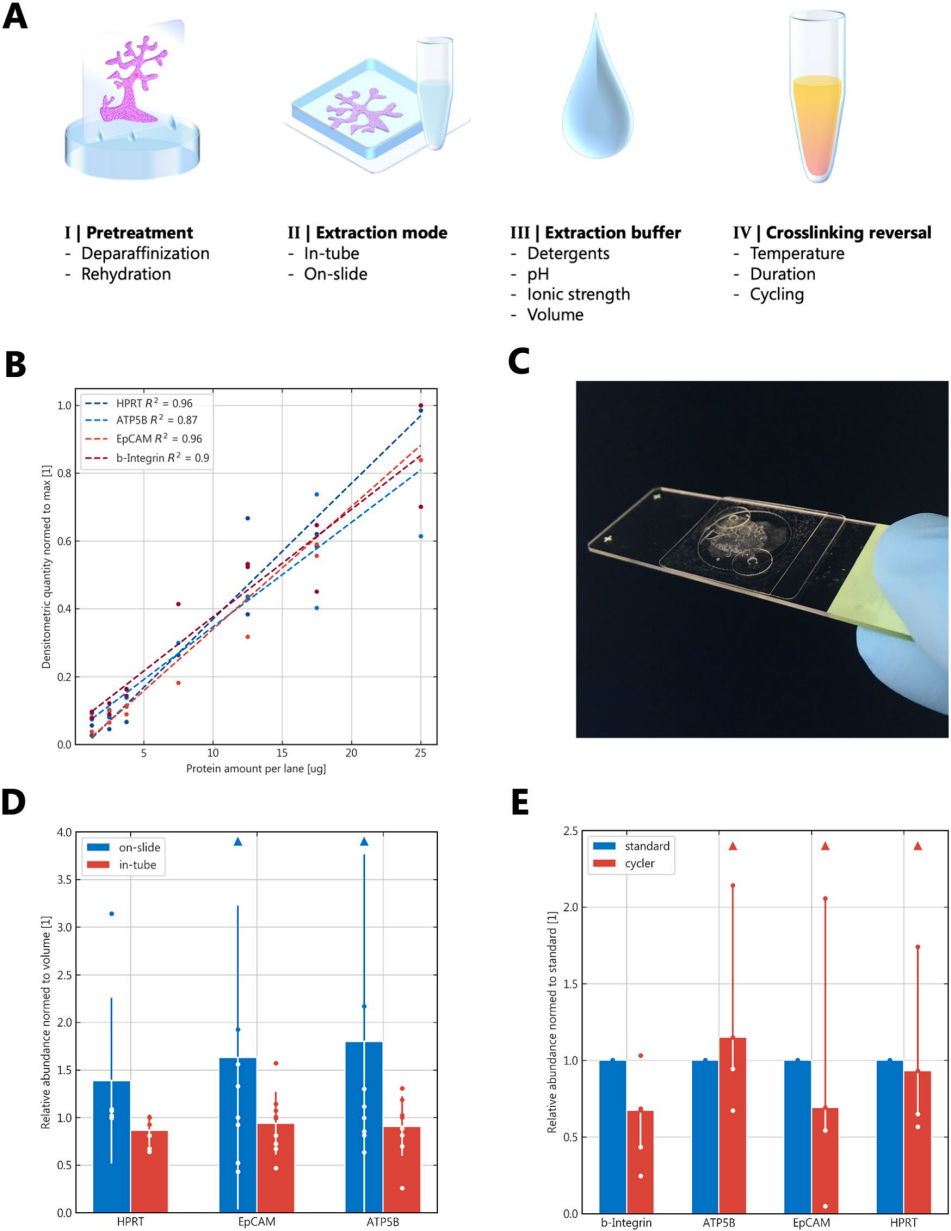
Evaluation setup and extraction mode*.* **A** Visual summary of the key aspects of FFPE protein extraction; **B** Dynamic ranges of the four marker proteins employed for western blot analysis; n = 2; **C** Exemplary image of a reaction chamber used for on-slide extraction. Triangles denote values outside of the visualization window; **D** On-slide extraction versus in-tube; n = 9, means ± standard deviation, normalized to median blot intensity to allow for visual variance comparison; **E** Comparison of the use of a thermocycler for heat-induced antigen retrieval compared to the standard protocol (identical temperature protocol); n = 5, medians with interquartile ranges of the respective data as whiskers

For the different optimization steps, parameter effects were determined by different measures: Protein concentration in the resulting lysate, mass spectrometric analysis of selected samples and with a standardized western blot assay to determine extraction bias and efficiency specific for the selected marker proteins. These were chosen to cover different cellular compartments and protein sizes and are listed in Table 1. To establish grounds for a quantitative evaluation, the signal-to-input relation was closely examined beforehand and was found to exhibit sufficient linear correlation (Pearson’s correlation coefficient 0.87–0.96). The resulting ranges and regressions are shown in Fig. 1B.

**Table 1:** Selected marker proteins for assessment of biased protein extraction

Compartment	Marker	Molecular size (kDa)	Isoeletric point
Cytosol	HPRT	23	6.24
Mitochondria	ATP5B	50	5.00
Plasma membrane	EpCAM	38	7.56
Plasma membrane	beta-Integrin	135	5.03–5.95

### Statistical notes

The number of biological replicates was set depending on the a prior importance and likelihood of change of a specific parameter as well as the limits given by the number of lanes for parallel western blot analysis. Cross-blot comparisons were avoided except for the evaluation of thermal effects, for which a common standard was necessary due to the wider variation. All results are shown with medians for replicates above four and means below, as the median implicitly handles deviant values as outliers at low sample size [43]. All values are reported as data points, accompanied by standard deviation and interquartile range (IQR) respectively. With this reporting scheme, we aim at providing the reader with a comprehensive and (relatively) unbiased view of the data and refrain from performing statistical tests that are likely to be rather misguiding than helpful in the present setting of low sample size and multiple comparisons.

### Extraction mode

The use of commercially available on-slide PCR reaction chambers (Fig. 1C) was successfully implemented for protein extraction. While marker protein quantity was comparable and even higher in some cases than in-tube extraction, handling proved to be more time consuming and less reproducible, mirrored by considerably increased standard deviation of the results (Fig. 1D).

**Fig. 2:**
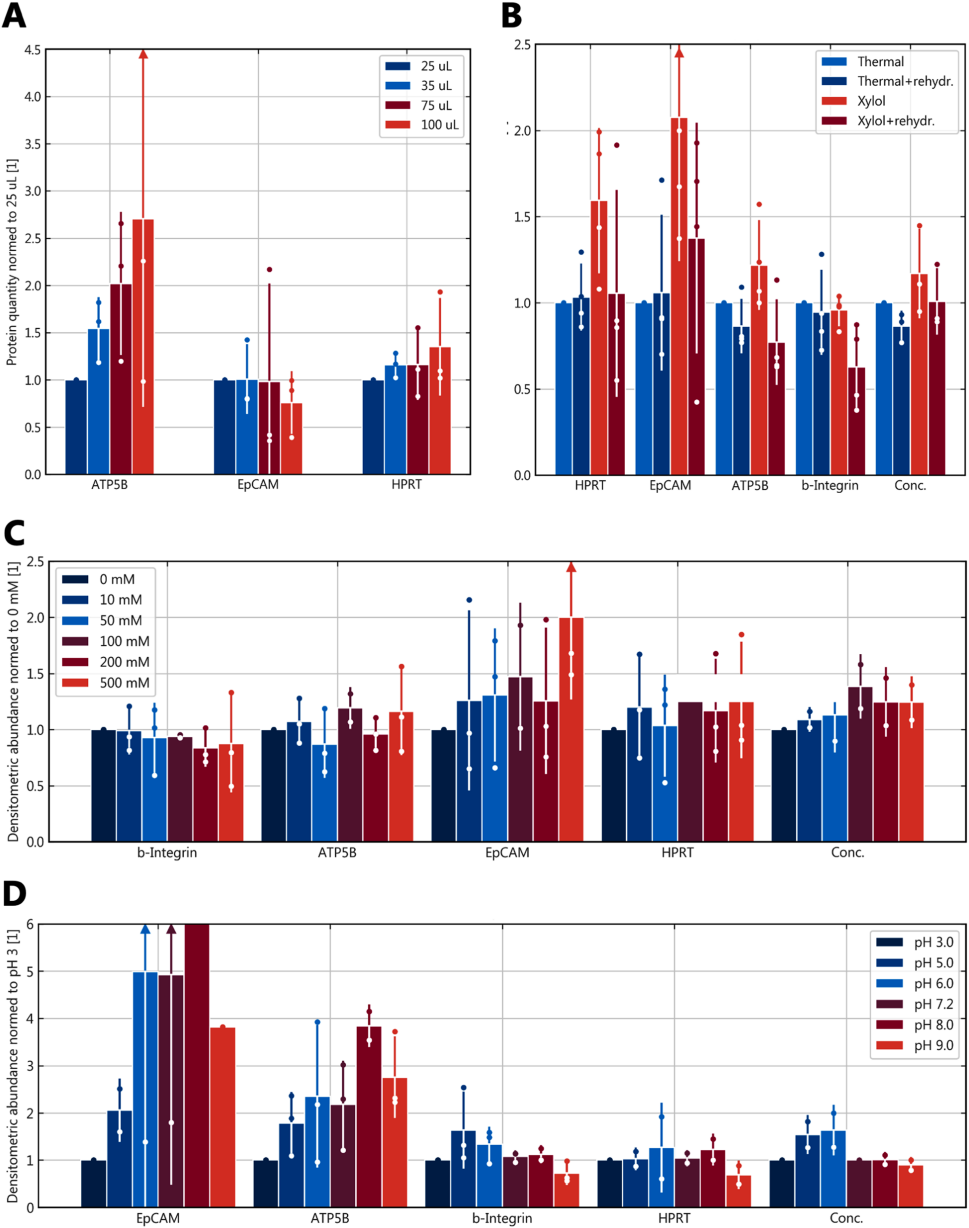
Physicochemical parameters and sample preparation. **A** Buffer volume variation and resulting overall marker quantity; n = 3, means ± standard deviation; **B** Effects of different deparaffinization and rehydratation regimens; n = 4, means ± standard deviation; **C** Effects of ionic strength variation on protein extraction efficiency; n = 3, means ± standard deviation; **D** Effects of pH variation in analogy to subplot A

Using a thermocycler proved to reduce hands-on/attendance time considerably from several minutes within 140 min to a single start of the cycler with the samples being held at 4 °C automatically upon completion. At the same time marker protein extraction was of comparable efficiency when compared in the standard temperature setting (100 °C for 20 min followed by 80 °C for 120 min; Fig. 1E). The optimal volume for protein extraction in-tube was found to be 100 µl (Fig. 2A).

### Deparaffinization

Deparaffinization was compared between thermal and xylol-based protocols. The results are shown in Fig. 2B and demonstrate some increase with xylene deparaffinization compared to thermal.

### Rehydratation

The addition of rehydratation rather reduced protein concentrations and quantities in Western blot analysis (Fig. 2B). LC–MS analysis comparing thermal to combined deparaffinization/rehydratation approaches demonstrated equal performance of the thermal protocol while fewer membrane proteins were identified when xylol/ethanol was used, similar to the western blot results (Additional file 1: Table S1).

### Ionic strength

Ionic strength was varied in an SDS-containing buffer by adding NaCl upto 500 mM in concentration. NaCl was chosen to avoid formation of precipitates with SDS and to use a neither strongly chaotropic nor strongly cosmotropic anion to prevent precipitation by either mechanism. There was no discernable effect of ionic strength variation (Fig. 2C).

### pH

Buffer pH is similarly linked to solubilization of proteins as overall charge and thus solubility vary with protonation and deprotonation of amino acids. Changing the buffer pH from 1 to 9 showed no relevant protein extraction at pH 1 (data not shown) while an optimum was reached at pH 6–8, so around the isoelectric point of most proteins (Fig. 2D) including the marker proteins (Table 1).

### Detergents

**Fig. 3:**
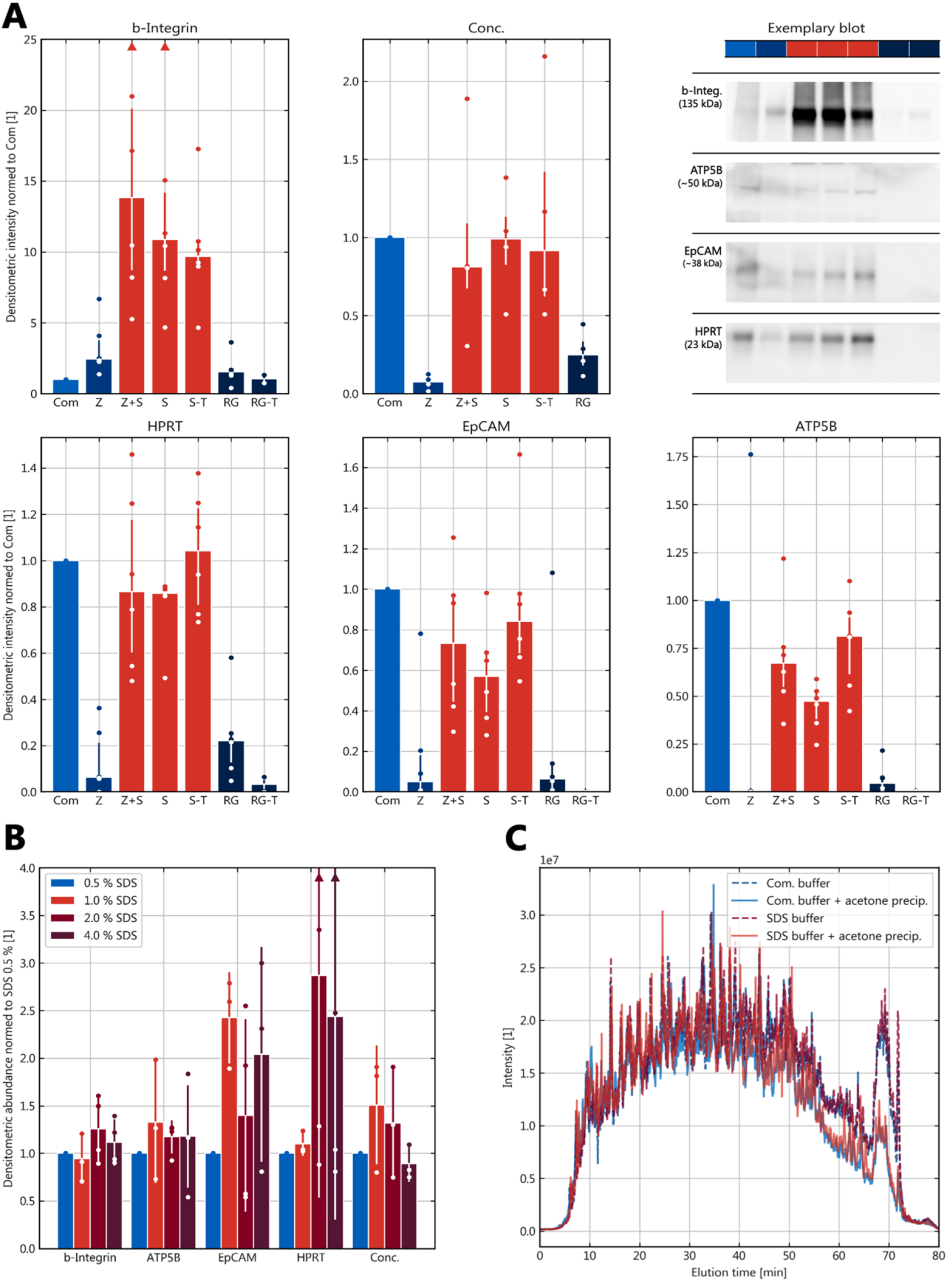
Buffer composition and detergents*.* Subplots report the different concentrations and quantities. **A** n = 6, medians with interquartile range of the respective data as whiskers; Com = commercial buffer EXB; Z = buffer containing Zwittergent 3-16; Z + S = buffer containing Zwittergent and SDS; S = buffer containing SDS only; S-T = buffer containing SDS and lower concentration of Tris; RG and RG-T = in analogy for RapiGest; please refer to the text for the detailed buffer compositions; **B** Effects of SDS concentration variation (w/v); n = 3, n = 4 for some proteins, means ± standard deviation; **C** High-performance liquid chromatography elution profile (total ion current) of sample preparation with buffer and precipitation variation for LC–MS

The effects of different detergents were investigated (Fig. 3A). While RapiGest (RG) and Zwittergent 3-16 containing buffers (Z) showed little extraction efficiency across proteins, the presence of SDS in combination with or without Zwittergent (Z + S) as well as at different ionic strengths and levels of Tris (S/S-T) showed comparable extraction efficiencies compared to the commercial qProteome buffer Com with only mildly reduced levels of ATP5B. The latter, however, showed markedly (> tenfold) decreased quantities of b-Integrin. With SDS being also included in the proprietary buffer Com, the level of SDS was varied (Fig. 3B), which showed an optimum for overall protein concentrations at 1–2% (w/v) SDS with similar effects on marker protein quantities. LC–MS of samples extracted with Com or buffer S* showed higher general identification rates of proteins as well as membrane proteins (Table 2) in favor of buffer S*, while chromatography elution profiles were highly comparable (Fig. 3C).

**Table 2:** Mass-spectrometric comparison of the two key buffers and precipitation modes

Buffer	Commercial (Com)		Custom (S*)	
Precipitation mode	Methanol	Acetone	Methanol	Acetone
Peptides identified	7595	6048	8634	8210
Proteins identified	1258	1064	1347	1361
Membrane proteins	164	124	184	167
% of all proteins	13.0	11.7	13.7	12.3
Cumul. intensity [arb.]	8.96 E + 07	6.60 E + 07	1.10 E + 08	1.01 E + 08
Median	1.34 E + 04	1.19 E + 04	1.44 E + 04	1.32 E + 04
Mean	7.12 E + 04	6.20 E + 04	8.19 E + 04	7.45 E + 04

**Fig. 4:**
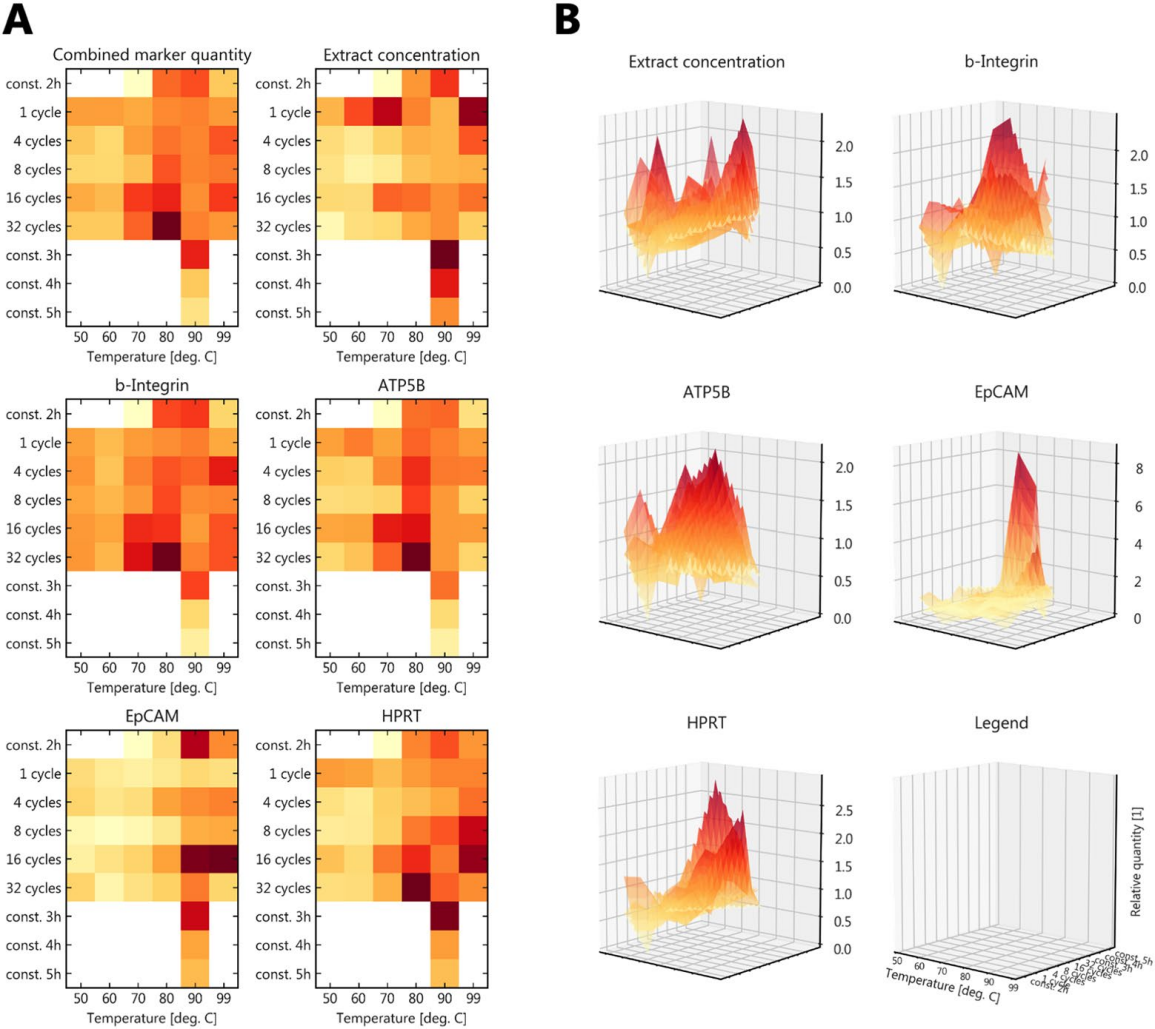
Temperature effects in heat-induced antigen retrieval. **A** Heatmaps showing the relative quantities and concentrations; distribution arrays were normed to the interval [0,1] with 0 = minimum (yellow) and 1 = maximum value (dark red) and averaged for n = 2 samples (n = 1 for constant 2 h, blot reproduced once); combined marker quantity averaged beta-Integrin, ATP5B and HPRT–EpCAM was not included due to much lower expression levels; **B** Quantitative differences of subplot A as overlay of sample-specific distributions (n = 2); samples were normed to their parameter-specific mean to enable comparison between replicates chosen to cover different levels of overall protein content; visualization by tenfold linear interpolation to improve visibility of color coding (0 = yellow, max = dark red)

Please see Fig. 4 for the respective total ion currents

### Formalin scavengers

Based on previously published data, the effect of elevated levels of Tris as scavenger molecule was examined (Fig. 3A). The substitution of HEPES with Tris at 200 mM to RG did rather decrease protein levels, an effect that was similarly observed in SDS-containing buffers with higher Tris concentrations (200 vs. 10 mM, S vs. S-T). The addition of amino acids as scavenger molecules did not improve antigen retrieval (Additional file 1: Fig. S2).

### Heat-induced antigen retrieval

The use of a thermocycler allowed for the standardized variation of temperature protocols. Figure 4A sums up the results, combined from multiple western blot analyses by use of a common standard. Figure 4B gives an idea of the actual quantitative variations implied, which range up to one-fold differences. While protein concentration is comparatively high even at low cycle numbers, marker-specific quantity increases with the number of heating cycles. Prolonged exposure to elevated temperature leads to reduction of both marker quantity and overall protein concentration.

### SDS removal

To simplify SDS removal and speed up the protocol, the methanol/chloroform precipitation was replaced by acetone precipitation, which proved equally effective (Table 2, Fig. 3C) but reduced manual steps from 16 to 4 and yielded a more stable pellet.

### Final comparison

**Fig. 5:**
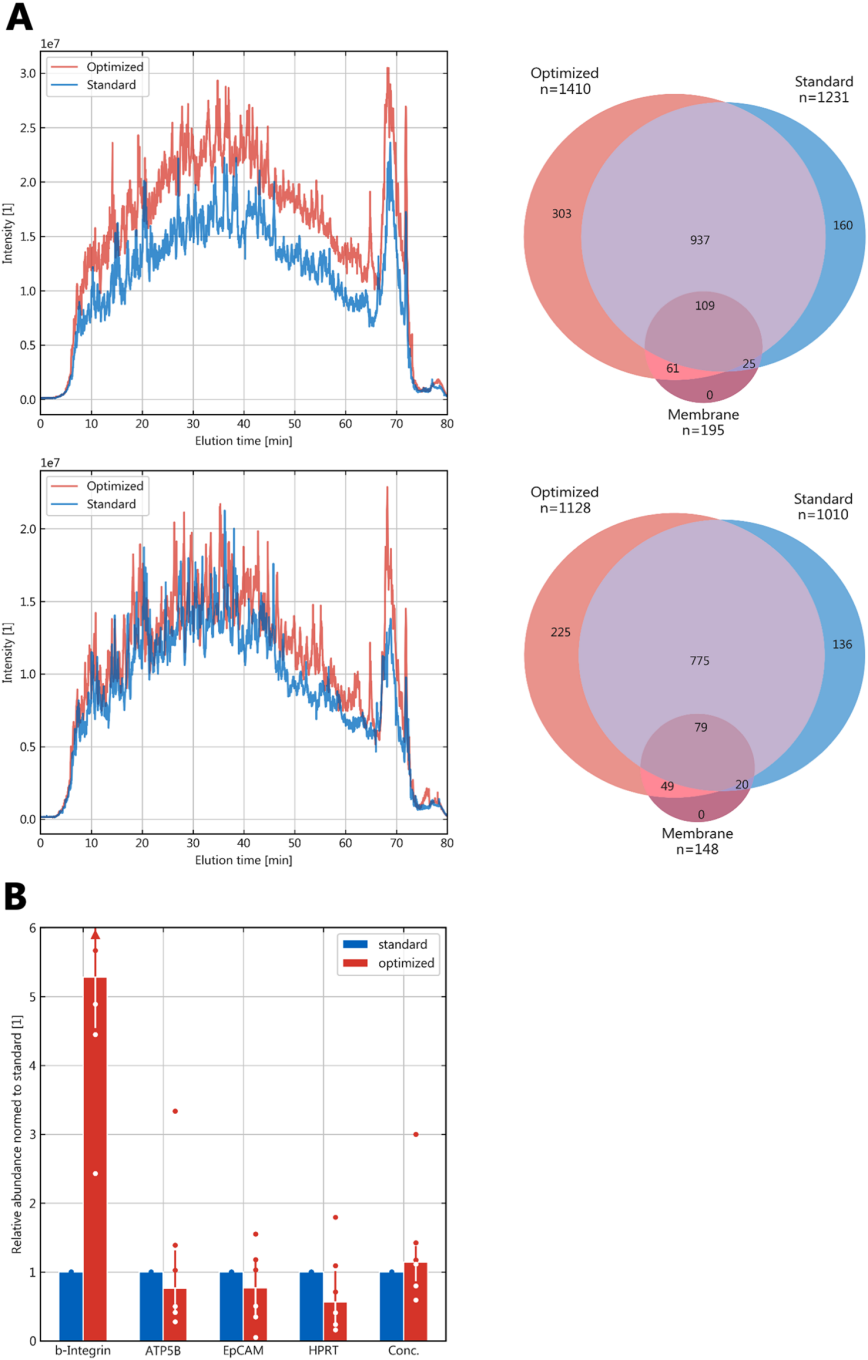
LC–MS comparison of the optimized versus the standard qProteome protocol*.* **A** Extracts from two biological replicates (different tissue blocks, different tumors) were analyzed (top and bottom row). (Left): Elution profiles (total ion current); (Right): Venn diagrams showing the distribution of the identified proteins with membrane proteins as separate subset; **B** Western blot comparison between the commercial qProteome protocol and buffer (standard) with the optimized protocol; n = 6, medians with interquartile ranges of the respective data as whiskers

The optimized protocol (Table 3) was compared to the standard qProteome protocol (Fig. 5). Western blot analysis showed comparable overall concentration and quantities of three out of four marker proteins (Fig. 5B). With the optimized protocol beta-Integrin was markedly increased by about four-fold of the qProteome level. LC–MS results showed similar differences between the qProteome and buffer S* with higher protein identification rates and shares of membrane proteins in samples that were extracted with buffer S* (Table 2). Comparing the complete optimized protocol to the standard qProteome protocol with LC–MS similarly demonstrated increased identification numbers and a higher share of membrane proteins (Fig. 5A): with the optimized protocol 14.5 and 11.7% more proteins were identified while MPs were overproportionally increased by 26.9 and 29.3% in the two biological replicates. The optimized protocol until sample clean-up required 10 min hands-on time in 35 min sample preparation with a subsequent break of variable length. The qProteome standard protocol required 20 min hands-on time in 4 h of sample preparation. The price per sample was about 95% lower with the custom buffer S*.

**Table 3:** Optimized protocol for protein extraction

1. Start with 2 tissue Sects. (10 µm) on glass slides or directly in tube
2. Incubate in xylol (100%) for 15 min
3. Discard xylol
4. Repeat once from step 2
5: Immerse briefly in ethanol (70 % v/v) to remove residual xylol
6. Immerse slides in double-destilled water for 30 s
7. Tap slides on paper towel to remove water, do not let dry
8. Scratch tissue into 0.2 ml PCR tube
9. Add 100 µl buffer S (SDS 2% (w/v), Tris-base 200 mM, EDTA 1 mM, pH 7.2) [µl]
10. Add 5 µl beta-mercaptoethanol [µl]
11. Add 1 µl proteinase & phosphatase inhibitor 1005 (ThermoFisher 78840) [µl]
12. Incubate on ice for 5 min
13. Vortex
14. Place tubes in a thermocycler:
15. 4 °C for 5 min
16. 90 °C for 90 min
17. 99 °C for 5 min
18. 60 °C for 10 min
19. Repeat 4 times from step 17 Step 16
20. Keep at 4° C if not processed directly
21. Vortex
22. Centrifuge at 4 °C, 10,000×*g* for 15 min
23. Save supernatant (extract)

## Discussion

FFPE tissue archives bear most of the biological information for actual translational research. Standardized protocols are necessary, especially on protein level, to ensure reproducibility. In the present study we systematically investigated the influence and relative effects of a variety of parameters. Special emphasis was put on the preservation and identifiability of membrane proteins as key proteins in many druggable oncogenic and pathological pathways.

### Optimized sample preparation

The use of optimal detergents has repeatedly been shown to be pivotal to protein extraction efficiency [44, 45]. In accordance with published data [25, 44], SDS showed high solubilization efficiency and exceeded RapiGest and Zwittergent considerably in our comparison. Simple SDS buffers also outperformed the widely used commercial EXB buffer in terms of extraction of membrane proteins. This has similarly been described by Nirmalan et al. [34] and is of high translational relevance, for instance in oncological biomarker screens, as actionable and prognostically relevant proteins are MPs overproportionally.

The importance of increased scavenger molecule concentration such as Tris could not be confirmed by our data. Close examination of the respective work by Kawashima et al. [15] revealed that the effect described appears to be more of a kinetic nature rather than altering quantitative endpoints. This could explain why such differences were not observed in the present study as we determined optimal extraction duration to be longer than in the respective publication.

Sample processing can further be streamlined and standardized by the use of thermal deparaffinization (as non-toxic alternative to xylol) and omission of time consuming rehydratation steps without negative impact on protein extraction efficiency, which was in part indicated in the data by Chung et al. [45]. Ionic composition has been shown to be of proteomic relevance, e.g. for increasing efficiency of acetone precipitation [46]. Varying ionic strength and pH did not reveal pronounced effects on protein extraction efficiency in our investigation but the parameter space is particularly wide for these factors and their interaction. Similar mixed results have been reported in heat-induced antigen retrieval protocols for IHC [17] with evidence hinting at an optimum at alkaline pH values, which we did not find.

Concerning sample clean-up strategies acetone and methanol/chloroform precipitation proved almost equivalent in LC–MS analysis with a considerably reduced number of steps and easier handling in favor of acetone precipitation, reducing technical variance. Concerning possible sample loss with precipitation methods, acetone precipitation has been shown to be close to complete when sufficient levels of sodium chloride or SDS are present [46]. With both added SDS and biological sodium levels in the samples exceeding the minimum value at least tenfold, further addition of sodium chloride is not necessary.

### Temperature effects

The use of a thermocycler has been mentioned in FFPE protein extraction before [47] but it was applied as substitute for a waterbath or heating block. Here, we report the application of a thermocycler to standardize heat-induced antigen retrieval in FFPE specimens while minimizing hands-on time and expanding the parameter space of possible temperature protocols. With the present study, we were able to cover combinations of temperature and duration from two to five hours and 60 to 99 °C as well as up to 32 temperature cycles. Our data suggests that a minimum of 70 °C has to be reached for antigen retrieval with an optimum around 90 °C. Exposure to elevated temperatures longer than 180 min tends to decrease extraction efficiency, most likely due to simple thermal degradation. With about one-fold quantitative differences we find evidence for increased marker extraction efficiency at a higher number of temperature cycles, while overall protein concentration seems to be less affected. This could root in the better separation of protein aggregates with repeated heating and cooling cycles leading to a better separation in SDS-PAGE and western blot analysis. Based on our data we recommend heating protocols that include temperature cycles but do not last longer than 180 min, avoiding prolonged exposure to temperature above 90 °C, for instance alternating between 90 and 70 °C for 16 cycles with a 1:2 distribution of temperature exposure and 140 min overall duration. During the course of our optimization, however, we have also gathered positive experience with a protocol combining constant exposure to 90 °C for 90 min and then alternating four times for 5 and 10 min between 99 and 60 °C, yielding consistent results across tissues and specimens (Additional file 1: Fig. S3; Table 3). Apart from optimizing antigen retrieval, which appears to differ to some extent with the marker protein, the advantage of using a thermocycler might be most evident in the mere standardization and consistent performance of the protocol independent of manual interaction.

### Conclusions

While several publications on individual parameters exist, we present a systematic approach providing a standardized read-out for variation of—at least to our knowledge—all a priori relevant parameters. Additionally, it is the first time that the effects of different temperature variations and automated temperature cycling for heat-induced antigen retrieval were systematically evaluated. We propose an optimized protocol for reproducible protein extraction from diagnostic FFPE tissue with simplified sample preparation to reduce non-biological variance. With the advent of faster and more sensitive mass spectrometers and data independent acquisition techniques the number of identifiable and quantifiable proteins has recently been considerably improved [48]. However, as demonstrated by our and other studies, the potentially biased influence of some parameters still exists on preanalytical level and may tamper with accurate quantitation—by both LC-MS or WB. We demonstrate that sample preparation can considerably be optimized in terms of protocol duration, standardization and cost effectiveness. Our protocol specifically addresses selective loss of protein subgroups and demonstrates balanced extraction performance in particular for the biologically highly relevant subset of membrane proteins.

## Supplementary Information

**Additional file 1: Method S1**. Stage-tip protocol. **Fig. S1**. Exemplary section of an FFPE urothelial cancer specimen. Asterisk marks interdigitating tumor cells within rich desmoplastic stroma, which makes extraction challenging; Hematoxylin-eosin stain, 20× magnification.** Fig. S2**: *Broadened scavenger spectrum by addition of amino acids during antigen retrieval/crosslink reversal. *Exemplary western blot showing unaltered extraction efficiency in various buffers (as in Fig. 3).** Fig. S3**: Extraction performance of the simplified buffer across tissue entities. Coomassie-stained SDS PAGE gel; 1 = pulmonary squamous cell carcinoma; 2 = pulmonary adenocarcinoma; 3/4 = prostate adenocarcinoma specimens; 5/6 = colorectal adenocarcinoma specimens; 40 % of maximum lane volume was loaded (corresponding to 20–40 μg per lane).** Table S1**. Mass-spectrometric comparison of different sample preparation protocols. X = xylol-based deparaffinization; E = ethanol-based rehydratation; H = heptane-based deparaffinization; M = methanol-based rehydratation.

## Data Availability

The data resulting from the current study are available from the corresponding author on reasonable request.
