# Supplementary material for: Systematic evaluation and optimization of protein extraction parameters in diagnostic FFPE specimens
Source: Clin Proteomics. 2022 May 2;19:10. doi: 10.1186/s12014-022-09346-0 (PMC9063121; doi:10.1186/s12014-022-09346-0)
Supplement: Supplementary file 1 — Additional file 1: Method S1. Stage-tip protocol. Fig. S1. Exemplary section of an FFPE urothelial cancer specimen. Asterisk marks interdigitating tumor cells within rich desmoplastic stroma, which makes extraction challenging; Hematoxylin-eosin stain, 20× magnification. Fig. S2: Broadened scavenger spectrum by addition of amino acids during antigen retrieval/crosslink reversal. Exemplary western blot showing unaltered extraction efficiency in various buffers (as in Fig. 3). Fig. S3: Extraction performance of the simplified buffer across tissue entities. Coomassie-stained SDS PAGE gel; 1 = pulmonary squamous cell carcinoma; 2 = pulmonary adenocarcinoma; 3/4 = prostate adenocarcinoma specimens; 5/6 = colorectal adenocarcinoma specimens; 40 % of maximum lane volume was loaded (corresponding to 20–40 μg per lane). Table S1. Mass-spectrometric comparison of different sample preparation protocols. X = xylol-based deparaffinization; E = ethanol-based rehydratation; H = heptane-based deparaffinization; M = methanol-based rehydratation. [file 12014_2022_9346_MOESM1_ESM.pdf]

– S U P P L E M E N T –

Systematic Evaluation and Optimization of Protein Extraction Parameters in Diagnostic FFPE Specimens

Supplemental method S1: Stage-tip protocol

Samples were dissolved in lysis buffer (25 mM sodium deoxycholate, 50 mM ammonium bicarbonate) and treated with ultrasonication. After centrifugation for 5 minutes at 10,000 g the supernatant was subsequently reduced and alkylated with 10 mM dithiothreitol and 10 mM iodoacetamide, respectively. Trypsin was added to the samples in a trypsin to protein ratio of 1:50 and incubated for 2 hours at 37 °C. Digestion was stopped by adding formic acid to a final concentration of 1%, lowering the pH to around 2. and precipitating the sodium deoxycholate, which was removed by centrifugation. Custom-made Stage-tips were prepared by punching out two layers of C8-membranes (solid phase extraction disk, Empore™, thickness 0.5 mm) and inserting them into a 200 µl pipetting tip. After preconditioning of the Stage-tip with 50 µl methanol and two times 100 µl aqueous triethylamine (0.1% v/v, pH 10), samples were added to the Stage-tip and centrifuged for 3 minutes at 3000x g. Samples were washed with 100 µl aqueous triethylamine for 3 minutes at 3000x g. Elution was performed in ten subsequent elution steps: 5 µl elution buffer (0.1% v/v aqueous triethylamine, pH 10) with increasing proportions of methanol (8%, 16%, 24%, 32%, 40%, 48%, 56%, 64%, 72%, 80%) were added to the Stage-tip and each fraction collected in a fresh tube by centrifugation for 3 minutes at 3000x g. The resulting 10 fractions per sample were vacuum dried and stored at -80°C until measurement.

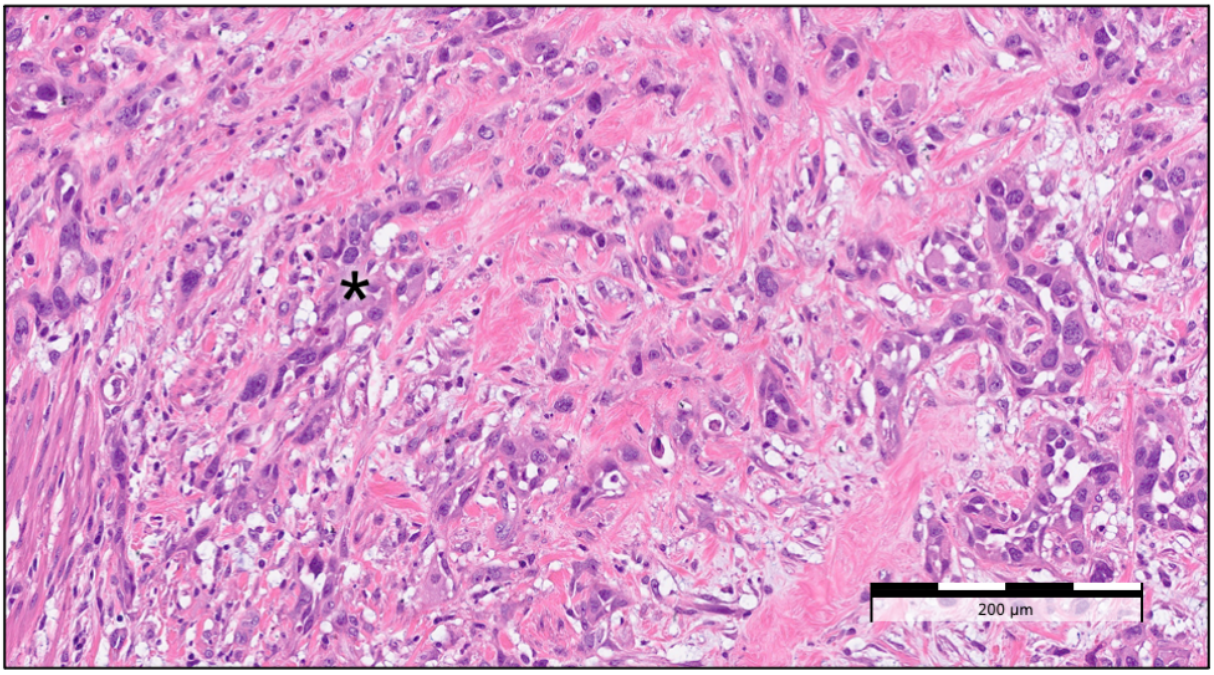

Fig. S1: *Exemplary section of an FFPE urothelial cancer specimen.* Asterisk marks interdigitating tumor cells within rich desmoplastic stroma, which makes extraction challenging; Hematoxylin-eosin stain, 20x magnification.

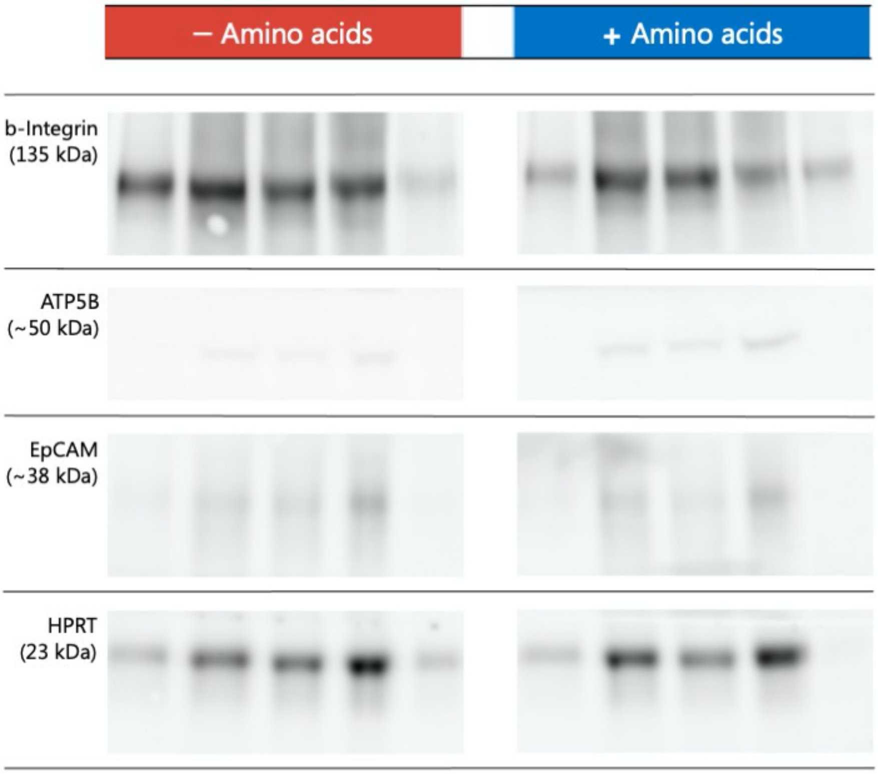

Fig. S2: *Broadened scavenger spectrum by addition of amino acids during antigen retrieval/crosslink reversal.* Exemplary western blot showing unaltered extraction efficiency in various buffers (as in fig. 3).

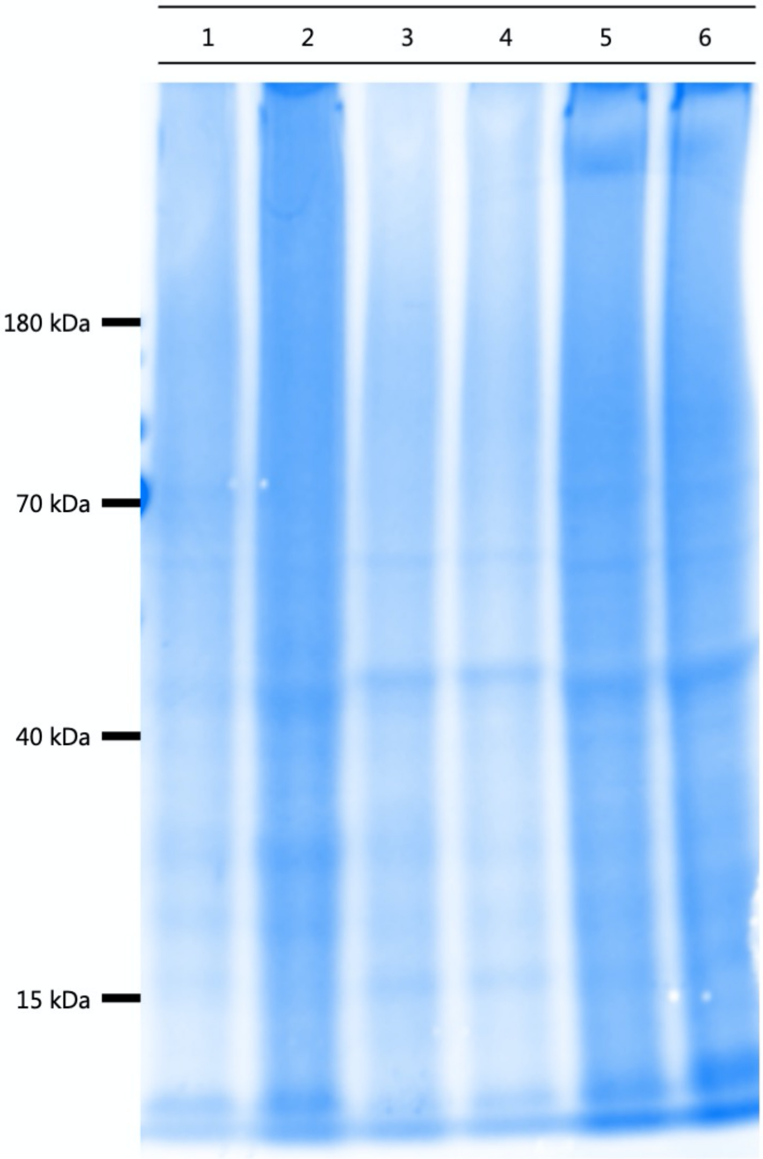

Fig. S3: *Extraction performance of the simplified buffer across tissue entities.* Coomassie-stained SDS PAGE gel; 1 = pulmonary squamous cell carcinoma; 2 = pulmonary adenocarcinoma; 3/4 = prostate adeno-carcinoma specimens; 5/6 = colorectal adenocarcinoma specimens; 40 % of maximum lane volume was loaded (corresponding to 20 – 40 µg per lane).

Digestion mode	In-gel			In-solution		
Deparaff./rehyd. mode	X+E	H+M	Thermal	X+E	H+M	Thermal
Peptides identified	7326	7228	7107	4227	5555	6347
Proteins identified	1459	1466	1467	1170	1425	1491
Membrane proteins	83	161	160	67	134	134
- % of all proteins	5.7	11.0	10.9	5.7	9.4	9.0
Cumul. intensity [arb.]	4.33 E+11	9.79 E+10	9.62 E+10	9.35 E+10	1.15 E+11	9.26 E+10
- Median	1.34 E+07	4.24 E+06	4.15 E+06	4.82 E+06	8.39 E+06	6.35 E+06
- Mean	2.97 E+08	6.68 E+07	6.56 E+07	7.99 E+07	8.10 E+07	6.21 E+07

Tbl. S1: *Mass-spectrometric comparison of different sample preparation protocols.* X = xylol-based deparaffinization; E = ethanol-based rehydratation; H = heptane-based deparaffinization; M = methanol-based rehydratation.

Buffer	Component	Concentration
Com	N/A	N/A
(commercial buffer	glycerol	1 – <10 % (w/w)
with PI)	SDS	1 – <10 % (w/w)
	2-ME	4.7 % (v/v)
	PI	0.9 % (v/v)
S*	SDS	2 % (w/v)
(final;	Tris-Base	200 mM
used also for pH	EDTA	1 mM
and ionic strength	pH	7.2
variation)	2-ME	4.7 % (v/v)
	PI	0.9 % (v/v)
RG	RapiGest™	0.1 % (w/v)
(from Foll et al.)	HEPES	100 mM
	DTT	1 mM
	pH	8
	2-ME	4.7 % (v/v)
	PI	0.9 % (v/v)
RG-T	RapiGest™	0.1 % (w/v)
	Tris-HCl	200 mM
	DTT	1 mM
	pH	8
	2-ME	4.7 % (v/v)
	PI	0.9 % (v/v)
S	SDS	8 % (w/v)
	Tris-Base	200 mM
	EDTA	1 mM
	pH	7.2
	2-ME	4.7 % (v/v)
	PI	0.9 % (v/v)
S-T	SDS	8 % (w/v)
	Tris-Base	10 mM
	EDTA	1 mM
	pH	7.2
	2-ME	4.7 % (v/v)
	PI	0.9 % (v/v)
Z	Zwittergent 3-16	2 % (w/v)
	Tris-Base	200 mM
	EDTA	1 mM
	pH	7.2

	2-ME	4.7 % (v/v)
	PI	0.9 % (v/v)
Z+S	SDS	8 % (w/v)
	Zwittergent 3-16	2 % (w/v)
	Tris-Base	200 mM
	EDTA	1 mM
	pH	7.2
	2-ME	4.7 % (v/v)
	PI	0.9 % (v/v)

Tbl. S2: *Buffer overview. DTT = Dithiothreitol; 2-ME = beta-Mercaptoethanol; PI = Phosphatase and proteinase inhibitor; SDS = Sodium dodecyl sulfate*
